# Transfemoral-Transcaval Liver Biopsy (TFTC) and Transjugular Liver Biopsy (TJLB) in Patients with Fontan-Associated Liver Disease (FALD)

**DOI:** 10.1007/s00270-024-03761-6

**Published:** 2024-05-30

**Authors:** Muhammad Usman Shahid, Yosef Frenkel, Norbert Kuc, Yosef Golowa, Jacob Cynamon

**Affiliations:** 1 Department of Interventional Radiology, University of Miami Miller School of Medicine, 1150 NW 14th Street, Miami, FL 33136 USA; 2 Division of Vascular and Interventional Radiology, Department of Radiology, Montefiore Medical Center, 111 E 210th St, Bronx, NY 10467 USA

**Keywords:** FALD, Fontan, Liver biopsy, Biopsy, Transjugular Liver Biopsy, Transfemoral Liver Biopsy

## Abstract

**Purpose:**

To describe our experience in performing transfemoral-transcaval liver biopsy (TFTC) and transjugular liver biopsy (TJLB) in patients with Fontan-associated liver disease (FALD).

**Methods:**

A single-center, retrospective review of 23 TFTC and seven TJLB performed between August 2011 and May 2023 on patients who previously underwent the Fontan procedure (median age 23.1 years, ranging 11–43 years, 48% female). Patient demographics, laboratory values, pathology, radiology, and cardiology reports were reviewed. Liver explants were correlated with histopathological evaluation to determine sampling accuracy when available.

**Results:**

All biopsies achieved technical success (accurate targeting and safe tissue sample extraction) and histopathological success (yielding sufficient tissue for accurate diagnosis). Liver biopsies were performed during simultaneous cardiac catheterization in 28 of 30 (93%) procedures. There was no statistically significant change in hemoglobin, hematocrit, platelet count post-procedure, and fluoroscopy times. There was one major complication within the TJLB group and one minor complication within the TFTC group.

**Conclusion:**

Transvenous liver biopsies, whether via transfemoral or transjugular route, may be safely performed in FALD patients while yielding samples with technical and histopathological success. The transfemoral approach, which is our preferred method; its compatibility with simultaneous cardiac catheterization and its potentially increased safety profile stemming from the avoidance of transversing the Fontan shunt—makes it a particular advantageous option in the management of FALD.

**Graphical Abstract:**

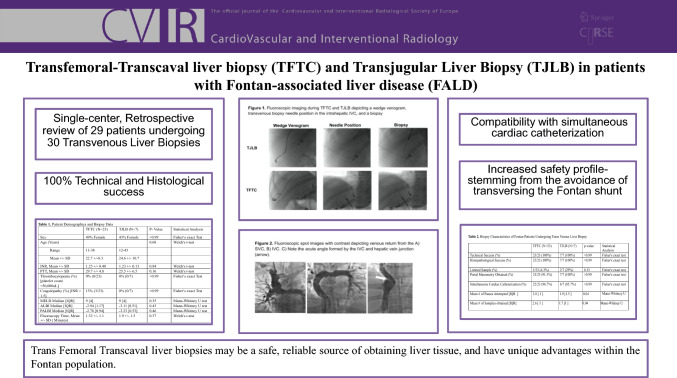

## Introduction

The Fontan procedure transformed the landscape for children with single-ventricle cardiac anomalies, marking a significant improvement in survival rates by redirecting venous blood directly to the pulmonary arteries [1, 2]. The Fontan procedure introduces complex changes to cardiovascular physiology by bypassing the right ventricle, creating a unique circulatory system where systemic venous return is passively directed to the pulmonary arteries. These physiologic changes boost 10-year survival rates but are also responsible for a new low cardiac output state and elevated central venous pressures, which can be 2–6 times baselines values that have detrimental effects on extracardiac organs such as the liver [3]. The elevated non-pulsatile venous pressures are directly transmitted to the hepatic veins and sinusoids, decreasing portal inflow leading to eventual fibrosis [4, 5]. This phenomenon is known as Fontan-associated liver disease (FALD). Liver biopsy remains the gold standard for liver disease assessment, and percutaneous liver biopsy poses unique challenges in Fontan patients, such as increased risk due their altered physiology and potential coagulopathies [3].

Transvenous liver biopsy can be performed in conjunction with the right heart catheterizations in coagulopathic patients while transfemoral-transcaval liver biopsy (TFTC) approach avoids transversing the Fontan shunt and a second venous access as cardiac catherizations are typically performed from a femoral approach while allowing for concurrent portal manometry. This retrospective single-center series presents our experience with TFTC biopsies in Fontan patients, exploring the safety and feasibility of this approach, and comparing it with the transjugular liver biopsy (TJLB) when TFTC is not feasible or contraindicated.

## Materials and Methods

This is a retrospective, institutional review board approved study, and informed consent was waived. Patients included in the study were identified via the Nuance PowerScribe 360 MONTAGE™ (Nuance, Burlington, MA) search tool. The electronic medical, pathological, and laboratory records of all patients who underwent transvenous liver biopsy post-Fontan procedure between August 2011 and May 2023 were reviewed. The start of the time frame represents the first case available for review in the electronic medical record. Coagulopathy and thrombocytopenia were defined using the SIR consensus thresholds of international normalized ratio (INR) of > 1.5 and the platelet count of less than 50,000 μL [6]. The number of patients who had stents placed prior to or during the procedure was recorded. Pre-biopsy liver function laboratory values were used to calculate model for end-stage liver disease (MELD), albumin–bilirubin (ALBI), and platelet–albumin–bilirubin (PALBI) scores. Patient demographics are summarized in Table 1.

**Table 1:** Patient demographics and biopsy data

	TFTC (*N* = 23)	TJLB (*N* = 7)	*P*-value	Statistical analysis
Sex	49% female	43% female	> 0.99	Fisher’s exact test
Age (years)			0.68	Welch’s *t*-test
Range	11–38	12–43		
Mean ± SD	22.7 ± 8.3	24.6 ± 10.7		
INR, Mean ± SD	1.25 ± 0.40	1.23 ± 0.13	0.84	Welch’s *t*-test
PTT, Mean ± SD	29.7 ± 4.8	25.5 ± 6.5	0.16	Welch’s *t*-test
Thrombocytopenia (%) [platelet count < 50,000 μL]	0% (0/23)	0% (0/7)	> 0.99	Fisher’s exact test
Coagulopathy (%) [INR > 1.5]	13% (3/23)	0% (0/7)	> 0.99	Fisher’s exact test
MELD median [IQR]	9 [4]	9 [4]	0.35	Mann–Whitney U-test
ALBI median [IQR]	− 2.94 [1.17]	− 3.11 [0.51]	0.43	Mann–Whitney U-test
PALBI median [IQR]	− 2.78 [0.94]	− 3.23 [0.53]	0.46	Mann–Whitney U-test
Fluoroscopy time, Mean ± SD (minutes)	1.32 ± 1.1	1.9 ± 1.5	0.37	Welch’s *t*-test

The pathologist documented the number of cores yielded by each case. The procedure was considered a technical success if hepatic tissue was successfully recovered, and histopathological success if the recovered sample was sufficient for accurate pathological diagnosis. Suboptimal or fragment sample was defined as a non-continuous 20-gauge 20-mm sample. When available, liver explant or additional biopsy pathology reports were reviewed and correlated with transvenous biopsy pathology reports.

Pre-procedural blood counts and coagulation parameters were routinely obtained before the procedure. Post-procedure, 2-h follow-up blood counts were obtained. Follow-up coagulation panels were not routinely obtained. Procedural complications were classified according to SIR guidelines [7].

### Technique

Transvenous liver biopsies were routinely performed simultaneously during cardiac catheterization for Fontan patients in our institution. Transvenous liver biopsies included both TJLB and TFTC. As described in Cynamon et al. [8], cross-sectional imaging or ultrasonography was reviewed prior to the procedure to evaluate the anatomy and course of intrahepatic inferior vena cava (IVC) and to confirm sufficient liver parenchyma. Sufficient liver parenchyma was defined as a length of 3 cm from the anticipated IVC puncture site. If cross-sectional imaging or ultrasonography was not available, then intraprocedural cone-beam computed tomography (CT) or intraprocedural ultrasonography was performed.

**Fig. 1:**
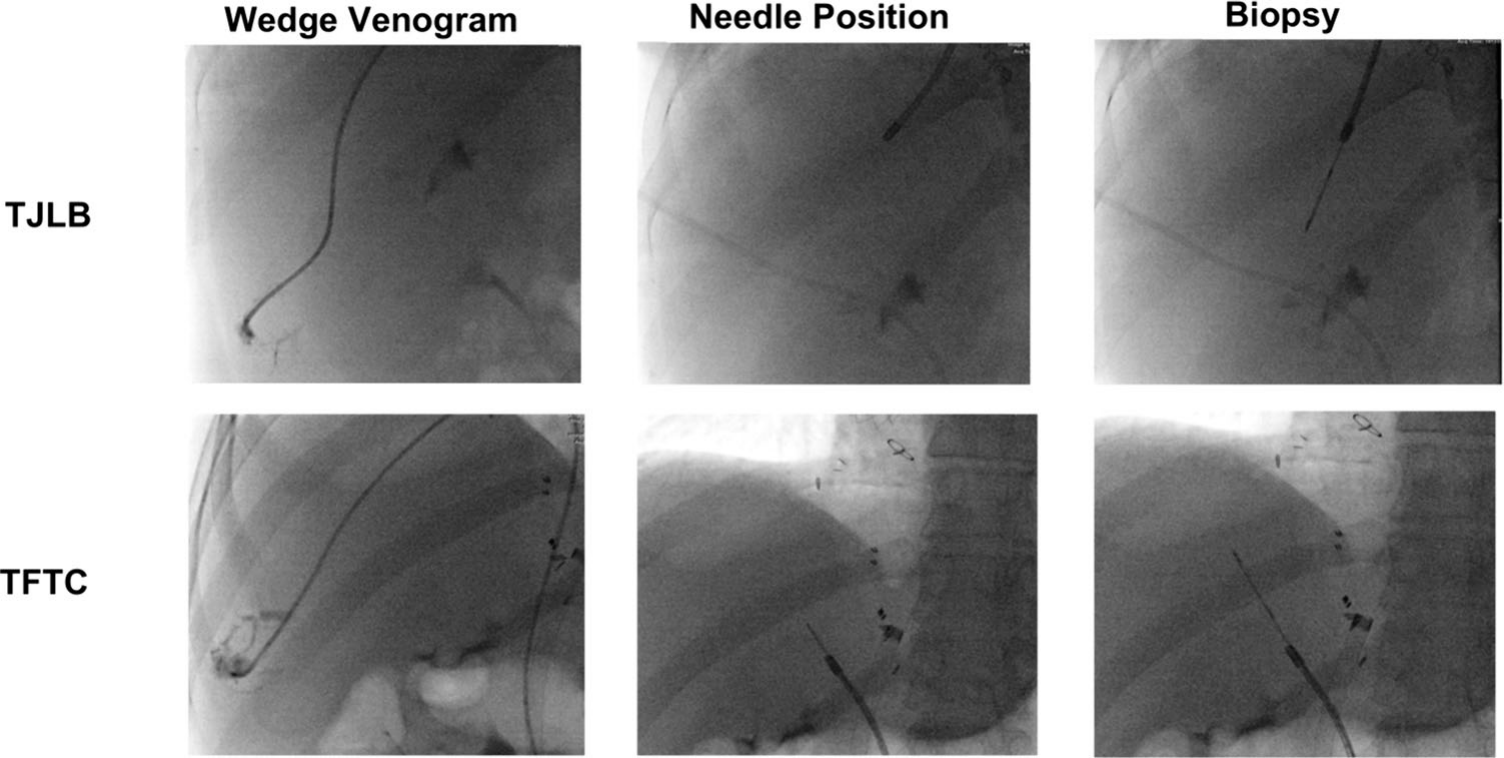
Fluoroscopic imaging during TFTC and TJLB depicting a wedge venogram, transvenous biopsy needle position in the intrahepatic IVC, and a biopsy

TFTC was conducted via existing access of the common femoral vein. The existing femoral sheath was exchanged for a 38.5-cm-long 10-F Sheath (Flexor Check-Flo II, Cook Medical, Bloomington, Indiana) and advanced over a 0.035-inch guidewire to the level of the hepatic veins. A 5-F Cobra (Cook Medical) or a 6-F Judkins Left-4 (Cook Medical) catheter was used to select a hepatic vein for portal manometry including wedged and free pressures. A physician-modified (increased curvature up to 45°) pre-curved 7-F stiffened cannula (Argon Medical Devices, Plano, TX) was advanced through a long femoral sheath over a guidewire and positioned approximately 1 vertebral body below the level of the hepatic veins. The 10-French sheath is retracted exposing the stiffened cannula. The physician-modified curve accentuates the curve up to 45° to achieve apposition of the tip against the lateral caval wall. The cannula is then directed toward the lateral caval wall within the intrahepatic IVC. Contrast is injected to confirm cannula wall opposition. Then, a 19-gauge biopsy needle with a 20-mm tray (Argon Medical Devices, Frisco, TX) was advanced through the lateral caval wall into the hepatic parenchyma under fluoroscopic guidance. The biopsy needle was subsequently fired under fluoroscopic observation to obtain the specimen (Fig. 1). Completion venogram was performed through the sheath to document the absence of caval injury/contrast extravasation.

TJLB was conducted via standard technique with Seldinger access of the right internal jugular vein. A 38.5-cm-long 10-French (Flexor Check-Flo II, Cook Medical) sheath was advanced over a 0.035-inch guidewire into the IVC. A 5-F Cobra (Cook Medical) or a 6-F Judkins Left-4 (Cook Medical) catheter was then used to select the right hepatic vein for portal manometry including wedged and free pressure. Next, the catheter was exchanged for the 7-F stiffened cannula (TLAB; Argon Medical Devices), which was advanced through the sheath and positioned in the right hepatic vein. Then, a 19-gauge biopsy needle with a 20-mm tray (Argon Medical Devices, Frisco, TX) was advanced through the hepatic vein wall into the hepatic parenchyma under fluoroscopic guidance.

For TFTC or TJLB, the attainment of at least one core was considered adequate tissue sampling. Patients were observed, and vital signs were monitored for at least 4 h. Post-operative CBC was performed two post-procedure routinely. No heparin was administered during the biopsy segment of the procedure. Biopsies were not performed which patients INR was greater than or equal to 3.0 or platelets under 20,000.

### Statistics

Statistical analysis was carried out using the GraphPad Prism 8 statistics program (GraphPad Prism Software, San Diego, CA). Categorical data were analyzed using Fisher’s exact test. Continuous variables with a normal distribution were analyzed using Welch’s *t*-test, and those with a non-normal distribution were analyzed with the Mann–Whitney U-test. Continuous variables were expressed as means except where a non-normal distribution was detected, in which cases, the medians were reported. *P*-values of less than 0.05 were considered statistically significant.

## Results

This study identified 29 patients undergoing 30 transvenous liver biopsies performed between August 2011 and May 2023. The median age of patients at the time of procedure was 23.1 years, ranging 11–43 years. Female patients comprised 48% of cases. Of the 30 biopsies conducted, 23 (77%) procedures were done via the TFTC approach and 7 (23%) via the TJLB approach. One patient underwent both transjugular and transfemoral liver biopsies 4 years apart. Liver biopsies occurred during simultaneous cardiac catheterization in 28 of 30 (93%) procedures. 7/30 (23%) of patients had prior stenting of the Fontan conduit, and 4 (14%) had stents placed during the procedure. Portal manometry was successfully obtained in all cases.

All procedures achieved technical and histopathological success, defined as attainment of at least one core biopsy sample, and the same was sufficient for an accurate pathological diagnosis, respectively. Three cases, 1/23 (4.4%) of TFTC and 2/7 (28.57%) of TJLB, returned suboptimal or fragmented samples as determined by the pathologist in which a pathological diagnosis was made and were considered successful.

For TFTC biopsies, the mean number of needle passes attempted was 2.8, and the mean number of specimens obtained was 2.6. For the TJLB group, the mean number of needle passes attempted was 1.9, and the mean number of specimens was 1.7. There was a statistically significant difference between the mean number of needle of passes attempted and samples obtained between the TFTC and TJLB groups (Table 2).

**Table 2:** Biopsy characteristics of Fontan patients undergoing transvenous liver biopsy

	TFTC (*N* = 23)	TJLB (*N* = 7)	*P*-value	Statistical analysis
Technical success (%)	23/23 (100%)	7/7 (100%)	> 0.99	Fisher’s exact test
Histopathological success (%)	23/23 (100%)	7/7 (100%)	> 0.99	Fisher’s exact test
Limited sample (%)	1/23 (4.3%)	2/7 (29%)	0.13	Fisher’s exact test
Portal manometry obtained (%)	21/23 (91.3%)	7/7 (100%)	> 0.99	Fisher’s exact test
Simultaneous cardiac catheterization (%)	22/23 (96.7%)	6/7 (85.7%)	> 0.99	Fisher’s exact test
Mean # of passes attempted [IQR]	2.8 [1]	1.9 [1.5]	0.04	Mann–Whitney U
Mean # of samples obtained [IQR]	2.6 [1]	1.7 [1]	0.04	Mann–Whitney U

Thrombocytopenia was not present in any TFTC or TJLB patients, and coagulopathy was present in 3 (13%) TFTC and 0 (0%) TJLB patients with an elevated INR above 1.5. There was no statistically significant difference in the decrease in hematocrit, hemoglobin, or platelet count post-biopsy between procedures (Table 3).

**Table 3:** Patient laboratories pre- and post-biopsy

	Pre-biopsy TFTC (*N* = 23)	Post-biopsy TFTC (*N* = 7)	*P*-value	Statistical analysis
Hemoglobin (g/dL) mean ± SD	15.02 ± 2.18	14.16 ± 2.67	0.35	Welch’s *t*-test
Hematocrit, mean ± SD	45.6 ± 5.81	42.96 ± 7.47	0.30	Welch’s *t*-test
Platelets (μL), mean ± SD	198.6 ± 85.54	162.75 ± 76.98	0.22	Welch’s *t*-test
	Pre-biopsy TJLB (*N* = 7)	Post-biopsy TJLB (*N* = 5)		
Hemoglobin (g/dL) mean ± SD	14.46 ± 2.93	12.9 ± 3.62	0.45	Welch’s *t*-test
Hematocrit, mean ± SD	43.14 ± 8.44	38.82 ± 10.44	0.47	Welch’s *t*-test
Platelets (μL), mean ± SD	174.43 ± 80.84	136.2 ± 67.85	0.40	Welch’s *t*-test

There was one major complication post-TJLB. The patient experienced a hypotensive episode post-biopsy with a hemoglobin drop from 11.5 to 7.0 g/dL. A non-contrast CT was performed of the abdomen which revealed a subcapsular hematoma and a hemoperitoneum. The patient was given a red blood cell transfusion, and hemoglobin improved to 10.1 g/dL prior to discharge 48 h later (SIR grade D major complication). There were no major complications following TFTC, but there was one minor complication. Six-h post-procedure, the patient experienced bleeding from the femoral venous puncture access site upon standing. Hemostasis was achieved with manual compression, and the patient was admitted for monitoring overnight. The patient was discharged 24 h later without additional intervention (SIR grade B minor complication).

## Discussion

FALD, an extra cardiovascular complication that is a major of morbidity, is detected universally upon biopsy for patients who undergo the Fontan procedure, ranging from mild fibrosis in the early stages of the disease to cirrhosis in the late stages [9]. Evaluating the extent of liver fibrosis is crucial, especially when considering further interventions including potential combined heart/liver transplants [10, 11]. Liver biopsy is accepted as the gold standard for assessing non-Fontan liver disease [12]; however, invasive evaluation of FALD is not well-studied. The paucity of studies on liver biopsies in FALD raises concerns that there may be a higher complication rate due to operator inexperience with complex multisystem diseases [3]. Additionally, studies show that congestive hepatopathy is less uniformly distributed within the liver than liver disease from other causes [13, 14]. Some authors advocate for percutaneous liver biopsy over transjugular liver biopsy (TJLB), which avoids sampling the hepatic venous wall and may yield higher quality samples [6]. However, percutaneous liver biopsy carries an increased risk for Fontan patients who can have coagulopathies secondary to liver dysfunction and limited cardiac reserve at baseline [3]. One study reported hemorrhage occurred in 7.4% of Fontan patients undergoing percutaneous liver biopsy, and another experienced an even higher bleeding incidence of 20.6% [15, 16]. Prior studies on TJLB in Fontan patients population have demonstrated safety as well as simultaneously being performed during routine surveillance cardiac catheterization [17]. The transvenous biopsy method may limit hemorrhage risk while allowing concurrent hemodynamic evaluation of the Fontan shunt as well as portal manometry.

At our institution, we follow the American Heart Association’s recommendation for invasive assessment of the Fontan shunt with cardiac catheterization every 10 years [18]. Our protocol includes acquiring a transvenous liver biopsy with portal manometry concurrently with cardiac catheter evaluation of the Fontan shunt. We have adopted the TFTC approach as our primary technique, which we believe offers several advantages in this unique patient population. This retrospective review assesses the utility of TFTC versus TJLB in Fontan patients. Peng et al. [19] demonstrated the safety and efficacy of TFTC compared to TJLB in the general population; however, there are no studies to date that assess these two transvenous biopsy techniques in the Fontan patient population. Our findings demonstrate that transvenous liver biopsies including TFTC are safe procedures that yield reliable results in patients with Fontan anatomy which allow for histopathological diagnosis and portal manometry concurrently with cardiac catheterization.

**Fig. 2:**
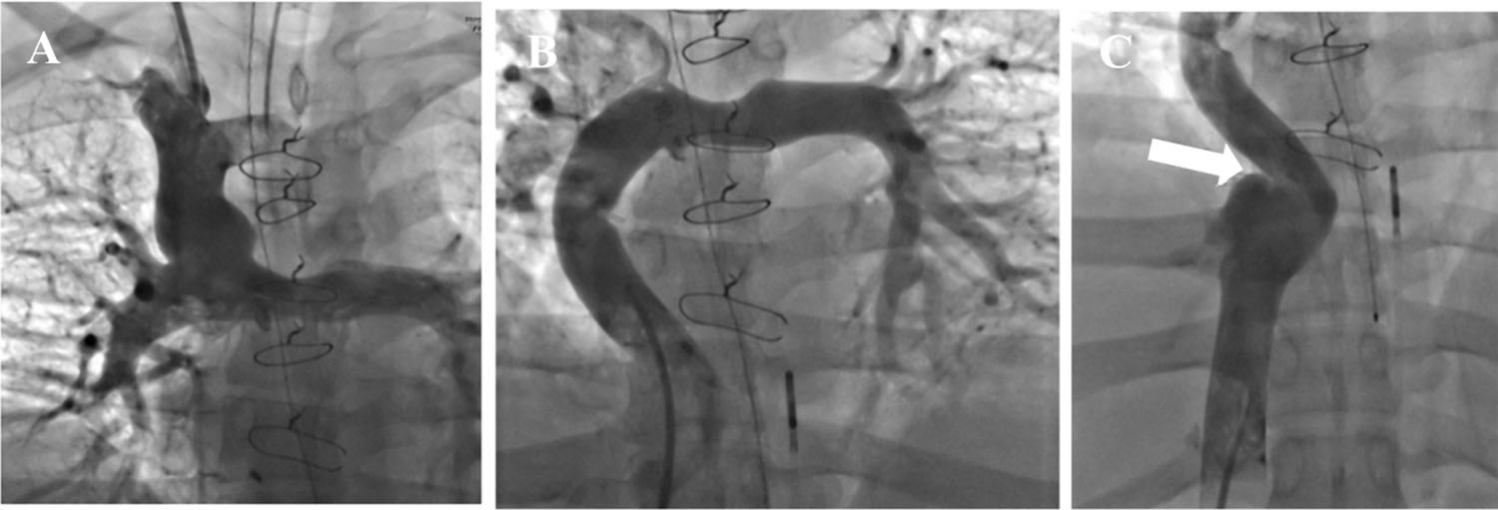
Fluoroscopic with contrast depicting venous return from the **A** SVC and **B** IVC. **C** Note the acute angle formed by the IVC and hepatic vein junction (arrow)

TFTC offers some potential advantages over conventional TJLB in this population. Firstly, TFTC avoids traversing the Fontan shunt and right pulmonary artery segment with a stiff cannula. The surgical anastomoses are not always aligned, and there may be severe angulations that preclude placement of the stiffened cannula into the inferior vena cava from a right internal jugular vein approach increasing procedural difficulty (Fig. 2). Secondly, the biopsies are often performed simultaneously with Fontan evaluation, and stents are frequently deployed in the shunt to decrease flow resistance. TFTC avoids traversing these stents, which may not be desirable.

Third, Peng et al. [19] demonstrated a lower overall complication rate of TFTC compared with TJLB, possibly because TFTC points the needle toward a segment of the liver that is less likely to contain the hepatic artery and portal vein. A study performed by Howaida G. El Said et al. who performed that transvenous TJLB performed in 125 Fontan patients and demonstrated a complication rate of 3.2% with 3 (2.4%) SIR grade D complications and 1 (0.8%) SIR grade B complication versus an SIR percutaneous liver biopsy threshold of 1.5% [20]. In our TFTC group, we did not experience any SIR grade D complications. These findings may suggest that TFTC may also be safer than TJLB for transvenous liver biopsies in the Fontan-specific population. In this study involving 29 patients undergoing 30 procedures, there were two hemorrhagic complications. One patient who underwent a TJLB and subsequently developed a subcapsular hematoma and hemoperitoneum resulting in a 4.5 g/dL hemoglobin drop (SIR grade D major complication). This may have been secondary to more central biopsy segment resulting in hepatic arterial injury [19]. The second hemorrhagic complication secondary to an access puncture site complication following TFTC liver biopsy upon standing (SIR grade B minor complication). This is speculated secondary to coagulopathy with an elevated INR above 1.5.

Fourth, Fontan patients have a higher risk of developing arrhythmias [21]. In patients with an intracardiac Fontan, the passage of a cannula through the right atrium can trigger an arrhythmia. The development of supraventricular arrhythmias is a known complication of TJLB and a potential cause of mortality [22–24]. TFTC avoids traversing this sensitive area in these certain subsegment of Fontan surgical subtypes and virtually eliminates this risk. Finally, when biopsies are performed during simultaneous cardiac catheterization, TFTC eliminates the need for secondary venous access when the primary access site is at the femoral vein. Femoral access has become the preferred method by the cardiologists performing cardiac catheterization within the Fontan population at our institution.

In our experience, the available explants and percutaneous biopsies were concordant with transvenous biopsies. This suggests that despite the well-documented heterogeneity of FALD, transvenous liver biopsies obtain representative samples, and it may be unnecessary to expose Fontan patients to potential increased risk of a percutaneous biopsy to acquire an accurate diagnosis. In our study, there was a slight non-statistically significant drop in hemoglobin, hematocrit, and platelet count that was likely a result of the hemodilution that occurs in transvenous procedures. There was also a statistically significant difference in between the mean number of needle passes attempted and the mean number of samples obtained between the TFTC and TJLB groups (Table 2). However, this is due to the increased number of needle passes attempted within the TFTC group in comparison with the TJLB group. All cases, however, achieved technical and histopathological success even in the setting of suboptimal or fragmented segments obtained in some cases.

There are several limitations to this study. This retrospective study is limited by selection bias which may be present as patients at our institution gradually transitioned from TJLB to TFTC over a 2-year time period due to perceived better technique of the procedure. Additionally, not all patients had post-biopsy laboratory studies, limiting the power of the post-biopsy comparisons. Different pathologists interpreted each biopsy sample, and inter-observer reliability for this interpretation was not known. Finally, this was a single-center study, limiting generalizability to other patient populations or operators. Not all cardiologists may choose to perform cardiac catheterization using femoral access, and this may be institution dependent. When cardiac catheterization is performed via the transjugular route, a secondary venous access point would be necessary to perform TFTC.

This study demonstrates that transvenous liver biopsies may be a safe, reliable source of obtaining liver tissue, and have unique advantages within the Fontan population. We recommend that transvenous liver biopsy be considered for Fontan patients and that the transfemoral route be considered as the primary transvenous approach.
